# A Frequency-Aware Self-Supervised Framework for MEMS-OCT Denoising

**DOI:** 10.3390/bios16030177

**Published:** 2026-03-21

**Authors:** Gaolin Zhang, Zonghao Li, Hui Zhao, Zhe Peng, Huikai Xie

**Affiliations:** 1 School of Integrated Circuits and Electronics, Beijing Institute of Technology, Beijing 100081, China; 2 LightVision Technologies Inc., Ltd., Foshan 528000, China; 3 BIT Chongqing Institute of Microelectronics and Microsystems, Chongqing 401332, China

**Keywords:** OCT denoising, self-supervised, frequency domain

## Abstract

Optical coherence tomography (OCT) is a key biological sensing and imaging tool widely used in biomedical detection, and its images are often degraded by multiplicative speckle noises—especially when micro-electro-mechanical system (MEMS) mirrors are employed in endoscopic OCT imaging, which reduces visual quality and affects the accuracy of subsequent analysis. Traditional denoising algorithms and supervised deep learning approaches have shown some effectiveness, but they are limited by their reliance on paired noisy–clean data and their insufficient modeling of global structural dependencies. To address these issues, this paper proposes a frequency-domain enhanced UNet based on the Neighbor2Neighbor (N2N) framework (FEN2N). The proposed FEN2N integrates wavelet-guided spectral pooling modules (WSPMs) and frequency-domain enhanced receptive field blocks (FE-RFBs). In this work, OCT images are obtained in a self-constructed MEMS-OCT system. Then the FEN2N is applied to the OCT image dataset. Results show that FEN2N achieves a more than 2.3 dB PSNR improvement over the N2N baseline, while the incorporation of FE-RFB contributes to a 0.02 improvement in SSIM. In addition, FEN2N outperforms several state-of-the-art methods, effectively suppressing speckle noise while preserving fine structural details that are important for clinical diagnosis.

## 1. Introduction

Optical coherence tomography [1] (OCT) is a non-invasive, high-resolution biological sensing and imaging modality widely used in ophthalmology [2], cardiology [3], and neuroscience [4]. Notably, OCT systems incorporating micro-electro-mechanical systems (MEMS) micromirrors for beam scanning enable system miniaturization, reduced driving requirements, and flexible two-dimensional scanning, which are beneficial for compact and portable implementations. Despite these advantages, the coherent nature of OCT detection leads to two major types of noise: high-frequency multiplicative speckle noise resulting from the interference of backscattered light from tissue microstructures, and low-frequency additive background artifacts induced by system-related instabilities, such as laser fluctuations and parasitic optical reflections. Together, these noise components degrade image quality and pose challenges to subsequent analyses, including microlesion identification and quantitative tissue assessment.

A broad array of specialized techniques have been dedicated to the issue of image denoising in MEMS-OCT imaging scenarios, yet existing denoising approaches still exhibit notable limitations. Traditional methods (e.g., BM3D [5,6]) rely on handcrafted priors that have limited adaptability to complex, signal-dependent OCT noise. Supervised deep learning [7] models (e.g., DnCNN [8,9], UNet [10,11]) depend on paired noisy–clean datasets, which are often difficult to acquire in practice. Self-supervised strategies (e.g., Noise2Noise [12], Noise2Void [13]) mitigate this dependence but often lack effective modeling of global dependencies and tend to cause over-smoothing. Extensions such as Noisier2Noise [14] introduce synthetic noise pairs yet remain limited by inaccurate noise modeling in real OCT data.

Recent studies have demonstrated that frequency-domain representation learning can effectively separate image structural features and noise components in the spectral domain, showing unique advantages in balancing noise suppression and structural preservation. Frequency-domain enhanced networks, such as wavelet-embedded UNet and spectral pooling-based denoising models, have been initially applied in natural image denoising and medical image segmentation tasks. However, existing frequency-domain denoising methods are mostly designed for Gaussian noise, and few studies have targeted the dual-type high/low-frequency noise characteristics of MEMS-OCT images. Meanwhile, the integration of explicit frequency-domain feature optimization and self-supervised denoising frameworks is still insufficient in existing research, which motivates our work.

Motivated by these insights, we propose FEN2N, a self-supervised denoising framework that integrates frequency-domain enhancement into the Neighbor2Neighbor (N2N) paradigm. Different from existing works, FEN2N leverages two core frequency-domain optimization modules derived from the classic FE-UNet framework [15]: the Wavelet-Guided Spectral Pooling Module (WSPM) and the Frequency-Domain Enhanced Receptive Field Block (FE-RFB). These two modules work synergistically to decompose OCT features into different frequency bands, dynamically fuse multi-scale spectral information, and effectively suppress noise while preserving critical tissue structural details. Specifically, WSPM applies Haar wavelet transform to decompose image features into different frequency subbands, which can suppress high-frequency multiplicative speckle noise while accurately preserving the effective high-frequency information that characterizes tissue edge and microstructure details; FE-RFB adopts a multi-branch dilated convolution structure to capture global low-frequency features, targeting the elimination of low-frequency background artifacts and improving the overall structural consistency of the denoised image. This design explicitly targets the dual nature of MEMS-OCT noise, while solving the core defect of existing frequency-domain methods that lose structural details while suppressing noise. The targeted contributions of the two modules are comprehensively verified by frequency-domain quantitative analysis and qualitative ablation visualization results in Section 4.3.2. The use of our MEMS-OCT [16] dataset—capturing diverse noise patterns and imaging artifacts—supports validation under realistic imaging conditions.

## 2. Preliminaries

### 2.1. MEMS-OCT System

MEMS refer to a class of miniaturized devices in which mechanical structures, sensors, actuators, and electronic components are fabricated and integrated using microfabrication technologies. Owing to their small size, low power consumption, and high mechanical precision, MEMS devices have been widely adopted in optical beam steering, inertial sensing, and biomedical instrumentation. In optical imaging systems, MEMS-based scanners have been extensively explored as alternatives to conventional macroscopic actuators, as they enable compact system architectures while maintaining precise and repeatable motion control [16]. These characteristics make MEMS well suited for portable and handheld imaging applications, where system size, weight, and stability are critical constraints.

The MEMS-OCT [17] system (SS-OCT) comprises three major functional components: a compact handheld imaging probe with 2D raster scanning capability, driven by an electrothermal MEMS actuator—realizing miniaturization (15 mm diameter, 40 mm length, 25 g weight) while ensuring precise scanning. An OCT interferometric module with a swept laser source (1310 nm, 100 nm bandwidth) and fiber-optic interferometry generates and detects depth-resolved interference signals. Additionally, a control and data processing unit coordinates MEMS scanning, high-speed signal acquisition, and image reconstruction, with external triggering ensuring synchronization between laser emission, scanning, and data collection.

**Figure 1:**
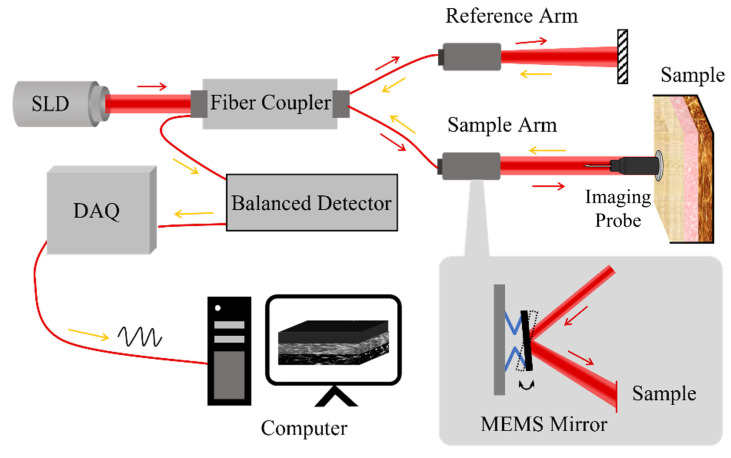
MEMS-OCT system. Arrows indicate the direction of light propagation. DAQ: data acquisition; SLD: super luminescent diode.

As illustrated in Figure 1, the swept laser source emits light split by a 2 × 2 fiber coupler into two beams: one travels to the reference arm, while the other is directed to the sample via the imaging probe (collimated, converged by a lens, and scanned by the MEMS mirror). Reflected/scattered light from the sample returns to form interference with the reference beam, which is captured by a balanced photodetector, transmitted to a computer via a high-speed DAQ card, and processed into 3D OCT images—aligning with the system’s portable and in vivo imaging design.

From the perspective of experimental data acquisition, MEMS-OCT provides several practical advantages. Owing to the compact design of the MEMS scanner, the imaging probe can be miniaturized, allowing handheld operation and in vivo imaging. At the same time, high-quality structural imaging is preserved, providing sufficient spatial resolution and penetration depth for the visualization of biological tissue microstructures. Furthermore, the system has demonstrated broad applicability across multiple imaging scenarios, including dermatological, endoscopic, and ophthalmic imaging, and has been successfully applied to both biological samples and human tissues.

The experimental image data were acquired using a self-developed MEMS-OCT system—a compact OCT implementation that replaces conventional galvanometer scanners with MEMS scanning technology is employed to replace conventional galvanometer scanners, thereby addressing the size and portability limitations of traditional OCT systems. While the intrinsic advantages of OCT, including non-contact operation, high spatial resolution, and millimeter-scale penetration depth, are maintained, MEMS-OCT enables flexible and portable imaging of biological tissues. In the present study, this system is utilized as the primary data acquisition platform.

### 2.2. Noise Analysis in MEMS-OCT

Despite these advantages, MEMS-OCT images are inevitably affected by hardware-related noise. Typical noise sources include multiplicative speckle noise inherent to coherent imaging, low-frequency background fluctuations induced by system instability, and coherent stripe artifacts arising from optical and electronic interference. The presence of these noise components degrades image quality and poses challenges for downstream quantitative analysis. These characteristics of MEMS-OCT data provide a strong motivation for the development of the proposed FEN2N denoising framework, which aims to suppress system-induced noise while preserving fine structural details in OCT images.

The proposed study is based on a self-developed MEMS-based OCT imaging system, designed for high-speed cross-sectional tissue acquisition. Although the system achieves stable axial resolution and broad spectral coverage, several hardware-induced imperfections introduce characteristic noise patterns that degrade image quality.

#### 2.2.1. Coherent Stripes

**Figure 2:**
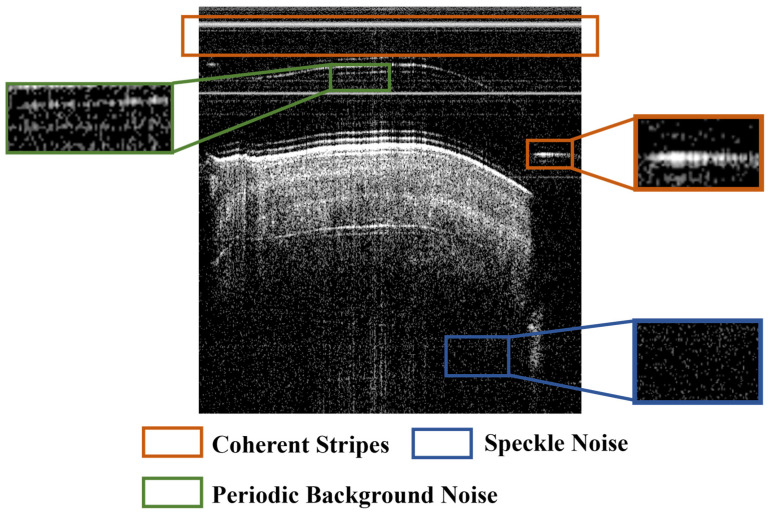
Three major noise types in images.

As indicated by the orange box in Figure 2, this noise is induced by Fresnel reflections between optical components in the interferometer. Such reflections produce spatially consistent horizontal or vertical stripe patterns that interfere with tissue visualization.

#### 2.2.2. Periodic Background Noise [18]

As marked by the green box in Figure 2, this noise arises from instability in the swept-source laser, detector nonuniformity, and MEMS mirror vibration. These factors lead to uneven illumination and low-frequency intensity bias across B-scans, often perceived as smooth background variation.

#### 2.2.3. Speckle Noise

As shown in the blue box in Figure 2, this noise is caused by the coherent interference of backscattered light from subcellular tissue microstructures. The random phase variations generate granular intensity fluctuations, obscuring fine anatomical details and reducing contrast.

These noise sources collectively result in a mixture of multiplicative and additive noise, presenting a major challenge for OCT denoising. An effective method must simultaneously suppress high-frequency speckle and low-frequency background noise while preserving structural fidelity.

To address these issues, we design the FEN2N framework, which explicitly targets each type of degradation through frequency-domain enhancement and self-supervised learning strategies.

## 3. Method

As analyzed above, OCT images exhibit both high-frequency speckle and low-frequency background noise. To address these issues, our method consists of three major stages: (1) artifact mitigation; (2) self-supervised pair generation; (3) FD denoising. Each component corresponds to a specific noise problem discussed in Section 3.

### 3.1. Dataset Preprocessing

The experimental data were collected using an optical coherence tomography (OCT) laser imaging system (LVM-1000, developed by LIVITECH, Foshan, China), equipped with a swept-source laser operating at 1060/1310 nm bands with a scanning frequency of 100 kHz, and a large-bandwidth, low-noise, high-speed acquisition card [19]. The following preprocessing steps were applied to the acquired images:

#### 3.1.1. Removal of Coherent Stripes

Coherent stripes, originating from Fresnel reflections of optical components in the system, were eliminated by leveraging their spatial consistency across consecutive B-scans. An image stacking strategy was employed to enhance tissue structures, followed by threshold-based segmentation to separate stripe artifacts from anatomical features.

#### 3.1.2. Removal of Periodic Background Noise

The noise characteristics were analyzed in the frequency domain via 2D Fourier transform. A customized notch filter was designed to attenuate dominant noise frequencies, and Gaussian blurring was applied to mitigate residual ringing artifacts.

**Figure 3:**
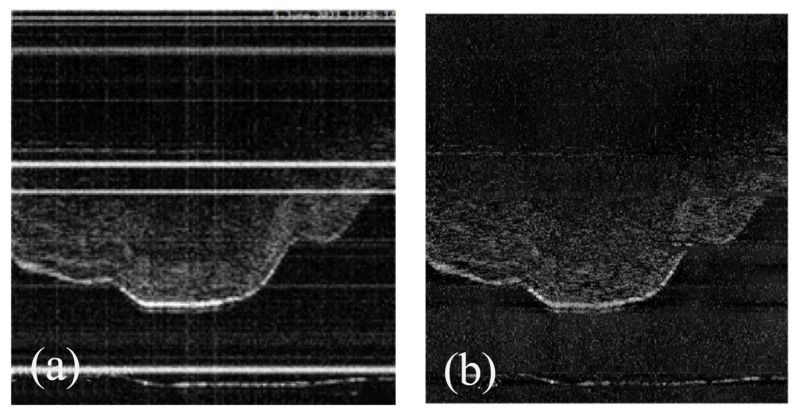
Preprocess. (**a**) Original. (**b**) Preprocessed.

These preprocessing steps mitigate dominant noise components, delivering an average 5–7 dB signal-to-noise ratio (SNR) gain for OCT images per the standard medical image SNR evaluation protocol, as illustrated in Figure 3. With residual noise further suppressed by the subsequent deep learning-based denoising framework.

### 3.2. Neighbor Subsampling

To avoid the reliance on paired noisy-clean data, we adopt the self-supervised N2N paradigm as the base sampling strategy, with tailored adaptations to match the noise characteristics of MEMS-OCT images.

As proposed, N2N relies on its ability to generate a pair of structurally consistent yet noise-distinct noisy image samples from a single noisy input via deterministic spatial subsampling, thereby obviating the requirement for multiple independent observations of the same scene. Specifically, by imposing non-overlapping adjacent spatial masking on a noisy MEMS-OCT image $y\in R^{H\times W}$ (where H and W denote the height and width of the image, respectively), two complementary subsampled image pairs $\left(g_{1}\left(y\right),g_{2}\left(y\right)\right)$ are derived. These subsampled images preserve the intrinsic structural coherence of the underlying tissue while minimizing noise distribution discrepancies, as the latent clean images they represent are semantically consistent. The training objective function of the original N2N framework is to optimize the reconstruction loss between the network-predicted denoised output of one subsampled image and the other subsampled image (treated as the pseudo-supervisory signal), which is mathematically formulated as:

(1) $$\underset{\theta }{\mathrm{argmin}}\;E_{x,y}{\left||f_{\theta }{(g}_{1}\left(y\right))-g_{2}\left(y\right)|\right|}_{2}^{2},$$

where θ denotes network parameters, x is the latent clean images, and $f_{\theta }$ represents the denoising network.

To adapt N2N to MEMS-OCT data—characterized by multiplicative speckle (high-frequency) and additive background noise (low-frequency)—we refine the subsampling strategy as follows:

#### 3.2.1. Structural Preservation Constraint

The spatial masks are designed to align with the axial resolution of MEMS-OCT (with a stable resolution of ~10 μm), ensuring that critical anatomical boundaries (e.g., tissue layers) are not split during subsampling.

#### 3.2.2. Noise Consistency Matching

Given the signal-dependent nature of OCT speckle, we limit the subsampling stride to 2 pixels, minimizing the variance of noise distribution between $g_{1}\left(y\right)$ and $g_{2}\left(y\right)$;

(2) $$\varepsilon =E_{y|x}\left(g_{2}\left(y\right)\right)-E_{y|x}\left(g_{1}\left(y\right)\right)\to 0,$$

#### 3.2.3. Regularization Adaptation

We retain N2N’s regularization term with γ = 0.01 (tuned via validation) to mitigate over-smoothing, which is particularly critical for preserving fine structural details (e.g., microlesion edges) in clinical OCT images.

The adapted subsampling strategy generates high-quality noisy image pairs, which serve as inputs for the subsequent frequency-domain denoising modules (WSPM and FE-RFB) to further separate noise and structural features.

### 3.3. Frequency-Domain Enhanced UNet

**Figure 4:**
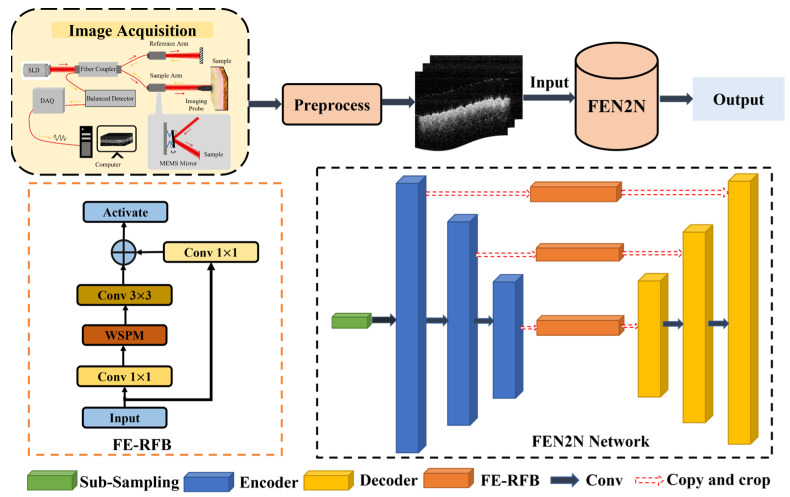
Structure of FEN2N network.

The proposed FEN2N framework innovatively incorporates a Frequency-Enhanced UNet (FE-UNet) architecture to address the dual-frequency characteristics of optical coherence tomography (OCT) noise. Specifically, OCT noise exhibits low-frequency structural distortions and high-frequency speckle artifacts, which pose significant challenges to image quality and subsequent analysis. The network architecture is illustrated in Figure 4.

#### 3.3.1. Encoder

The encoder of FE-UNet adopts a 4-layer hierarchical down-sampling structure and systematically integrates wavelet-guided spectral processing and multi-scale frequency fusion within the encoder–decoder framework, which is designed to extract hierarchical image features while effectively handling noise at different frequencies.

Wavelet-Guided Spectral Pooling Module (WSPM): Embedded in each branch of FE-RFB, this module applies Haar wavelet transform to partition input features into four subbands (LL, LH, HL, HH) representing different frequency components. We set the spectral mixing parameter λ to 0.65 to suppress high-frequency speckle noise while enhancing low-frequency tissue structural features, balancing the frequency distribution of extracted features [20].

Frequency-Domain Enhanced Receptive Field Block (FE-RFB): This module adopts a multi-branch dilated convolution structure to capture multi-scale complementary frequency information. Each branch uses dilated convolution to expand the receptive field without spatial resolution loss, and its output is refined by the embedded WSPM for joint spatial-frequency-domain optimization. This design adapts to the multi-scale distribution of biological tissue structures in OCT images and specifically suppresses low-frequency background artifacts caused by MEMS mirror vibration and laser source instability.

#### 3.3.2. Decoder

The decoder of FE-UNet adopts a symmetric 4-layer upsampling structure, which receives frequency-refined features from the encoder’s FE-RFB via skip connections, to restore image details while preserving frequency-domain information integrity [21]. For feature upscaling, a Frequency-Aware Upsampling Module adopts bilinear interpolation combined with 3 × 3 convolutions, which effectively mitigates grid artifacts that may occur during the upsampling process and ensures a smooth transition of features between different scales. The overall algorithm flowchart is shown in Algorithm 1.

**Algorithm 1:** Frequency-Domain Denoising

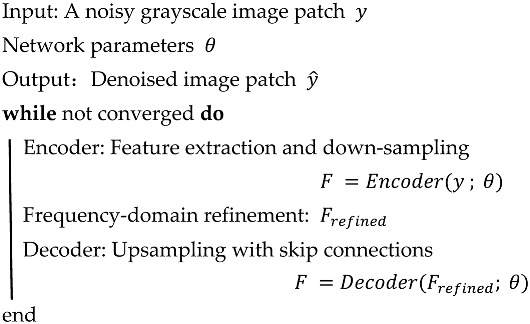



### 3.4. Self-Supervised Loss Function

By applying the theoretical framework established above, we derive and simplify the following expressions:

(3) $$L_{total}=\lambda_{1}L_{1}+\lambda_{2}L_{2}+\lambda_{3}L_{freq},$$

Sub-image Prediction Loss:

(4) $$L_{1}={||f}_{FE-UNet}^{*}\left(g_{1}\left(y\right)\right)-g_{2}\left(y\right)|{|}_{1},$$

Noise Distribution Consistency Loss:

(5) $$L_{2}={||f}_{FE-UNet}^{*}\left(g_{1}\left(y\right)\right)-f_{FE-UNet}^{*}\left(g_{2}\left(y\right)\right)|{|}_{2}^{2},$$

Frequency-Domain Consistency Loss:

(6) $$L_{freq}=||F\left(f_{FEN2N}\left(y\right)\right)-F(x_{pseudo})|{|}_{2}^{2},$$

where F is the 2D Fourier transform, $f_{FEN2N}\left(y\right)$ is denoised image output and $x_{pseudo}$ is the pseudo-clean image generated by neighbor subsampling. We set $\lambda_{1}=$ 0.8, $\lambda_{2}=$ 0.1, and $\lambda_{3}=$ 0.1 via grid search optimization on the validation set of the self-constructed dataset: the search range of each hyperparameter is set to [0.0, 1.0] with a step size of 0.1, and the parameter combination with the highest SSIM is selected since structural similarity is the key metric for evaluating clinical OCT image denoising. The regularization term γ  = 0.01 of the N2N framework is also optimized via the same grid search method to ensure the rationality and reliability of all hyperparameters. $L_{1}$ ensures pixel-level restoration accuracy; $L_{2}\;$ constrains consistent noise distribution between paired samples to avoid over-smoothing; $L_{freq}$ guarantees frequency-domain consistency between denoised images and pseudo-clean images, preserving structural fidelity.

## 4. Experiment

To validate the effectiveness of FEN2N for OCT denoising, we design comprehensive experiments covering baseline comparison, module ablation, and clinical downstream task verification. The following sections detail the experimental setup, results, and analysis.

### 4.1. Setup

Our FEN2N is an end-to-end trainable model without any pretrained networks. The network was trained for 100 epochs using the Adam optimizer with default beta parameters ($\beta_{1}$ = 0.9, $\beta_{2}$ = 0.999) and an initial learning rate of 1 × 10^−4^, with a batch size set to 4 and the learning rate halved every 20 epochs. The model was implemented in Python 3.7.1 with PyTorch 1.13.1, using an NVIDIA GeForce Titan RTX 24G GPU.

### 4.2. Datasets

The dataset is derived from a self-collected MEMS laboratory dataset, comprising 3472 finger tissue images and randomly split into training, validation, and test sets in an 8:1:1 ratio.

### 4.3. Result

Since this work adopts the self-supervised N2N framework, paired noisy-clean ground truth is unavailable for real clinical MEMS-OCT images due to practical acquisition limitations. Thus, the pseudo-clean ground truth used for calculating SNR, PSNR and SSIM is generated by the neighbor subsampling strategy described in Section 3.2: the original noisy image is split into two complementary subsampled images via deterministic non-overlapping adjacent spatial masking, where one is used as the network input and the other serves as the pseudo-clean ground truth for performance evaluation. This masking strategy is not random; we predefine a fixed non-overlapping adjacent pixel partitioning rule for MEMS-OCT images with fixed resolution, and the mapping relationship to generate complementary subsampled pairs ($g_{1}\left(y\right),g_{2}(y$)) is kept completely constant throughout the entire training, validation and test process. Based on the inherent property of strong spatial correlation in biological tissue images, which forms the foundational basis for the core assumption of the N2N framework, the subsampled pairs share highly consistent structural features and noise distributions, thus justifying the use of subsampled images as pseudo-clean ground truth. We have further optimized this sampling strategy for MEMS-OCT data in Section 3.2 by introducing three core constraints: structural preservation constraint, noise consistency matching and regularization adaptation. These constraints further compensate for minor pixel area differences between the subsampled images. The original N2N study and its subsequent derivative studies in medical image denoising have verified that this deterministic design ensures the uniqueness and stability of performance metric calculations, and the calculated metrics do not depend on the selection of subsampled ground truth. This label generation method is widely accepted and applied in self-supervised medical image denoising research, which ensures the rationality and comparability of quantitative metric calculation.

#### 4.3.1. Baseline Comparison

**Figure 5:**
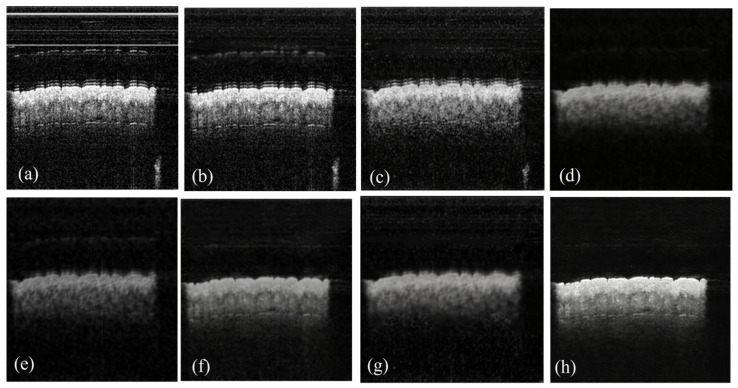
Denoised result. (**a**) Original. (**b**) Noisy. (**c**) Mean Filter. (**d**) DnCNN. (**e**) Noise2Noise. (**f**) Noise2Void. (**g**) N2N (Baseline). (**h**) Ours.

**Figure 6:**
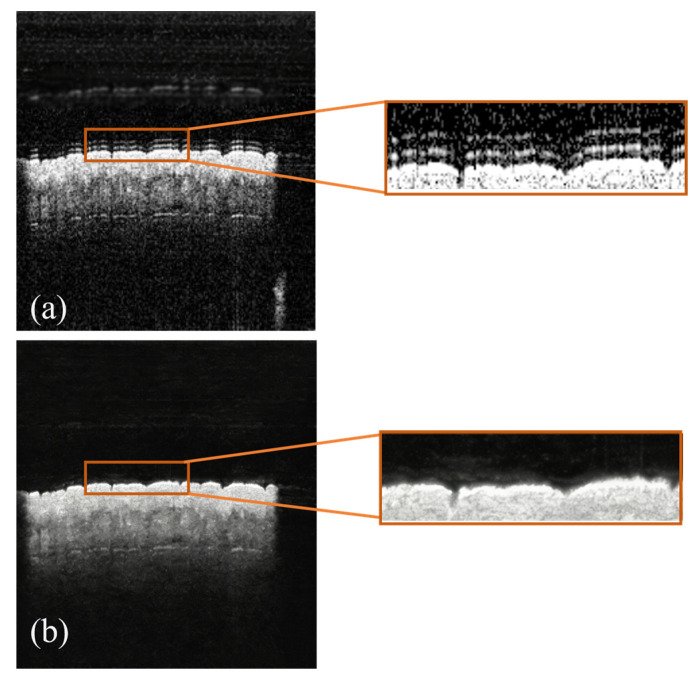
Details. (**a**) Noisy. (**b**) Ours.

To comprehensively evaluate the denoising performance of the proposed FEN2N, we select five representative methods for comparison, including traditional filtering, supervised deep learning, and self-supervised denoising frameworks. FEN2N achieves superior denoising performance, as demonstrated by higher SNR, Peak Signal-to-Noise Ratio (PSNR), and Structural Similarity Index (SSIM) metrics (Table 1). The performance analysis of each comparison method is as follows:

The baseline comparison results demonstrate the superiority of our FEN2N for MEMS-OCT denoising. Our method achieves the optimal performance in SNR (31.29 dB), PSNR (33.26 dB) and SSIM (0.9303) among all compared methods. Specifically, it brings a 1.65 dB SNR improvement over the N2N baseline, 0.0067 SSIM improvement over the Noise2Void, and outperforms the traditional The Mean Filter and the DnCNN model, both in noise suppression and structural detail preservation. Notably, the supervised DnCNN model causes structural damage with an SSIM even lower than the original noisy image, while our method effectively avoids this over-smoothing defect. The visual denoising results of all methods are shown in Figure 5, and the detailed results are presented in Figure 6.

#### 4.3.2. Ablation Experiments

**Figure 7:**
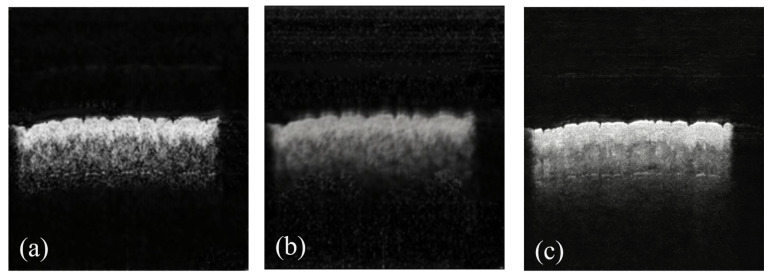
Qualitative ablation results of FEN2N. (**a**) w/o WSPM. (**b**) w/o FE-RFB. (**c**) Full.

Ablation results (Table 2) confirm the targeted contributions of WSPM and FE-RFB for MEMS-OCT image denoising and structural preservation: removing WSPM leads to a 2.4 dB SNR drop and a 0.0176 SSIM decrease compared with the full model. This performance degradation is not only due to the ineffective suppression of high-frequency speckle noise, but also because the network loses the ability to distinguish between high-frequency noise and effective high-frequency structural information, resulting in blurred tissue edges and loss of microstructure details. Removing FE-RFB causes a 0.0191 SSIM decrease and a 1.65 dB SNR drop, which is due to the loss of multi-scale dilated convolution-based global low-frequency feature capture capability, resulting in residual low-frequency background artifacts, blurred tissue layer boundaries and reduced overall structural consistency of the image. All variants are trained under the same experimental setup to ensure a fair comparison, and the qualitative denoising effects of each ablation variant are shown in Figure 7.

Benefiting from the synergistic integration of WSPM and FE-RFB, the proposed FEN2N outperforms traditional filtering methods and the N2N baseline in both quantitative metrics (SNR, PSNR, SSIM) and visual quality, effectively eliminating mixed noise in MEMS-OCT images while preserving clinically critical microstructures. Ablation experiments confirm the necessity of both modules, as removing either leads to significant performance degradation under unified training settings for fair comparison. As an end-to-end trainable model without relying on pretrained networks, FEN2N achieves a balanced enhancement in denoising efficacy and structural fidelity via its frequency-domain synergistic mechanism, providing a reliable solution for MEMS-OCT imaging and laying a solid foundation for subsequent clinical applications.

## 5. Conclusions

This paper presents FEN2N, a self-supervised denoising framework for MEMS-OCT that integrates wavelet-guided spectral pooling (WSPM) and frequency-domain enhanced receptive field blocks (FE-RFB). The framework addresses both high-frequency speckle and low-frequency background artifacts through a neighbor subsampling strategy, eliminating the reliance on paired clean-noisy data. Key contributions include a targeted frequency-domain analysis guiding speckle suppression, the integration of frequency-aware modules into an N2N backbone for adaptive feature balancing, and significant visual-quality improvement achieved by reducing artifacts while preserving critical anatomical details. Quantitatively, FEN2N outperforms existing methods, achieving an average PSNR gain of more than 2.3 dB and a 0.02 SSIM improvement over the N2N baseline. It effectively suppresses coherent stripes, periodic noise, and speckle, enhances image contrast and edge definition, and demonstrates strong potential for portable MEMS-OCT applications.

Future work will focus on extending the utility of FEN2N in medical image processing: integrating it as a preprocessing module to support downstream tasks such as tissue segmentation and lesion diagnosis, adapting the framework to multi-modal medical images by optimizing frequency-domain modules, and exploring lightweight network designs to facilitate its deployment on edge devices for clinical translation [22].

## Figures and Tables

**Table 1:** Quantitative result of the dataset.

	SNR (dB)	PSNR (dB)	SSIM
Original	20.47	22.32	0.8631
Noisy	25.62	26.11	0.8747
Mean Filter	27.79	29.85	0.8951
DnCNN	26.23	28.76	0.8587
Noise2Noise	28.58	30.81	0.9134
Noise2Void	27.88	29.13	0.9236
N2N (Baseline)	29.64	30.96	0.9112
Ours	31.29	33.26	0.9303

**Table 2:** Ablation experiment results.

	SNR (dB)	PSNR (dB)	SSIM
w/o WSPM	28.89	30.87	0.9127
w/o FE-RFB	29.64	30.96	0.9112
Ours	31.29	33.26	0.9303

## Data Availability

No new data were created or analyzed in this study.
